# Establishing Quality Indicators and Implementation Priorities for Post‐Stroke Aphasia Services Through End‐User Involvement

**DOI:** 10.1111/hex.14173

**Published:** 2024-09-02

**Authors:** Kirstine Shrubsole, Marissa Stone, Dominique A. Cadilhac, Monique F. Kilkenny, Emma Power, Elizabeth Lynch, John E. Pierce, David A. Copland, Erin Godecke, Bridget Burton, Emily Brogan, Sarah J. Wallace

**Affiliations:** ^1^ Queensland Aphasia Research Centre The University of Queensland Brisbane Australia; ^2^ Surgical Treatment and Rehabilitation Service (STARS) Education and Research Alliance The University of Queensland and Metro North Brisbane Australia; ^3^ Centre of Research Excellence in Aphasia Recovery and Rehabilitation La Trobe University Melbourne Australia; ^4^ Speech Pathology Department Princess Alexandra Hospital, Metro South Health Woolloongabba Queensland Australia; ^5^ Stroke and Ageing Research, Department of Medicine, School of Clinical Sciences at Monash Health Monash University Clayton Victoria Australia; ^6^ Stroke Division, The Florey Institute of Neuroscience and Mental Health University of Melbourne Heidelberg Victoria Australia; ^7^ Faculty of Health University of Technology Sydney Sydney Australia; ^8^ College of Nursing and Health Sciences Flinders University Adelaide Australia; ^9^ School of Allied Health, Human Services and Sport La Trobe University Melbourne Australia; ^10^ School of Medical and Health Sciences Edith Cowan University Joondalup Australia; ^11^ Sir Charles Gairdner Hospital Perth Western Australia Australia

**Keywords:** aphasia, evidence‐based practice, implementation, minimum data set, quality of care, stroke

## Abstract

**Background:**

Currently, there are no agreed quality standards for post‐stroke aphasia services. Therefore, it is unknown if care reflects best practices or meets the expectations of people living with aphasia. We aimed to (1) shortlist, (2) operationalise and (3) prioritise best practice recommendations for post‐stroke aphasia care.

**Methods:**

Three phases of research were conducted. In Phase 1, recommendations with strong evidence and/or known to be important to people with lived experience of aphasia were identified. People with lived experience and health professionals rated the importance of each recommendation through a two‐round e‐Delphi exercise. Recommendations were then ranked for importance and feasibility and analysed using a graph theory–based voting system. In Phase 2, shortlisted recommendations from Phase 1 were converted into quality indicators for appraisal and voting in consensus meetings. In Phase 3, priorities for implementation were established by people with lived experience and health professionals following discussion and anonymous voting.

**Findings:**

In Phase 1, 23 best practice recommendations were identified and rated by people with lived experience (*n* = 26) and health professionals (*n* = 81). Ten recommendations were shortlisted. In Phase 2, people with lived experience (*n* = 4) and health professionals (*n* = 17) reached a consensus on 11 quality indicators, relating to assessment (*n* = 2), information provision (*n* = 3), communication partner training (*n* = 3), goal setting (*n* = 1), person and family‐centred care (*n* = 1) and provision of treatment (*n* = 1). In Phase 3, people with lived experience (*n* = 5) and health professionals (*n* = 7) identified three implementation priorities: assessment of aphasia, provision of aphasia‐friendly information and provision of therapy.

**Interpretation:**

Our 11 quality indicators and 3 implementation priorities are the first step to enabling systematic, efficient and person‐centred measurement and quality improvement in post‐stroke aphasia services. Quality indicators will be embedded in routine data collection systems, and strategies will be developed to address implementation priorities.

**Patient and Public Contribution:**

Protocol development was informed by our previous research, which explored the perspectives of 23 people living with aphasia about best practice aphasia services. Individuals with lived experience of aphasia participated as expert panel members in our three consensus meetings. We received support from consumer advisory networks associated with the Centre for Research Excellence in Aphasia Rehabilitation and Recovery and the Queensland Aphasia Research Centre.

## 1 Introduction

Aphasia is an acquired language disorder characterised by impaired expression and comprehension in verbal and written modalities [1]. Approximately one‐third of stroke survivors are affected by aphasia [2]. Compared to stroke survivors without communication disability, people with post‐stroke communication impairment such as aphasia incur greater healthcare costs [3], receive worse quality of care [4] and experience poorer health [5] and health‐related quality of life outcomes [6]. A targeted response to improve care and outcomes for people with post‐stroke aphasia is needed.

Despite the existence of high‐quality clinical practice guidelines [7] and best practice statements [8] to guide practice, there are evidence‐practice gaps in aphasia services. These gaps occur across the continuum of care [9, 10], including in areas such as assessment [11], provision of tailored information [12] and treatment dose and duration [13]. Organisational constraints and process barriers are key contributors to these aphasia evidence‐practice gaps [14, 15], including a lack of routine performance measurement against best practice recommendations [16]. Routine audit and feedback—where services actively seek to modify practice following feedback that clinical care is not meeting desirable targets—is an effective implementation strategy in stroke care [17, 18] and healthcare more broadly [19]. It has been shown to optimise patient outcomes and enhance alignment with guideline recommendations [17, 20] and is, therefore, a likely solution to address known barriers and improve aphasia care.

In Australia, registries such as the Australian Stroke Clinical Registry (AuSCR) [21] permit timely and reflexive responses to service‐level variations in care quality; however, no aphasia‐specific data are currently collected. To date, the measurement of aphasia service quality has relied on bespoke surveys of specific practices, in specific contexts, at single time points [22, 23, 24]. Such results quickly become outdated, and self‐reporting bias may result in an overestimation of performance [25, 26]. Furthermore, survey data are aggregated across many sites and services, limiting opportunity for individual services to identify and respond to their own practice gaps. Overall, the lack of routine, specific, and up‐to‐date knowledge of the evidence‐practice gaps in aphasia care means that healthcare services cannot easily identify or respond to variations in care quality. Services do not have data to advocate for change and cannot determine which gaps should be prioritised to improve practice.

To measure the quality of aphasia care and identify the highest priority evidence‐practice gaps, there is first a need to determine which areas of aphasia practice are most important to recipients and providers of healthcare services (i.e., people with aphasia, their family members and health professionals) [27]. Consultation and collaboration with clinical ‘experts’ in research is a key principle of an integrated knowledge translation approach [28] that facilitates buy‐in [29] and can increase implementation success [30]. The involvement of both people with lived experience of stroke and health professionals has proven feasible for identifying implementation priorities for stroke [27], although this was not specific to aphasia. Though authors of a previous scoping review identified implementation priorities for aphasia based on a set of implementation criteria [9], this process did not involve prospective input from people with lived experience of aphasia or health professionals.

To enhance reliable and relevant monitoring of post‐stroke aphasia care, it is necessary to prioritise recommendations and develop a prioritised minimum data set of quality indicators for routine measurement. Input from people with lived experience and health professionals is essential for the minimum data set to be meaningful. This will enable efficient and person‐centred service improvement and support targeted implementation efforts to address identified gaps. Therefore, we aimed to:

a. identify important and evidence‐based recommendations for post‐stroke aphasia care;
b. establish multistakeholder consensus on a set of quality indicators to support implementation efforts through routine performance measurement; and
c. determine priorities for implementation of best practice in post‐stroke aphasia care.

## 2 Methods

### 2.1 Study Design

**Figure 1:**
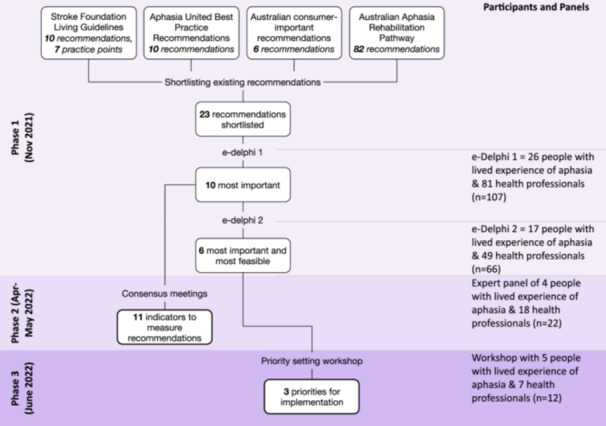
Flowchart of research phases, study timeline and participants or panel member details per phase.

This study was conducted in three phases to address each of our three aims (Figure 1). It comprised a two‐round e‐Delphi exercise, three consensus meetings and a priority‐setting workshop. People with lived experience of aphasia, clinicians and researchers were involved across all three phases, both as participants in consensus processes and as expert panel members. Throughout our research, the inclusion of people with aphasia was supported through the use of communication strategies provided by trained speech pathologists and the provision of communication‐accessible written information. Ethical approval was obtained from The University of Queensland Human Research Ethics Committee (approval number 2021/HE000735).

### 2.2 Phase 1a: Review, Selection and Shortlisting of Best Practice Recommendations

Members of the project team (authors K.S., E.P., E.L., J.E.P. and S.J.W.) adapted Lynch et al.'s [27] prioritisation process to identify and shortlist best practice recommendations. In our study, the aforementioned project team had expertise in implementing aphasia and/or stroke guidelines and developed the following criteria to prioritise recommendations based on Shrubsole et al. [9]: (i) strength of the evidence underpinning the recommendation, (ii) clinical importance as judged by people with lived experience and health professionals, (iii) feasibility of implementing the recommendation, (iv) health impact and (v) presence of known evidence‐practice gaps and/or ability to measure practice. A comprehensive list of internationally sourced evidence‐based aphasia recommendations was extracted (by author K.S.) from the Australian Aphasia Rehabilitation Pathway (AARP) best practice recommendations [8] and the Australian and New Zealand Stroke Clinical Guideline for Stroke Management (CGSM) [31]. The AARP has 82 best practice statements and is one of the most comprehensive aphasia guidelines available worldwide, whereas the Australian and New Zealand CGSM is the highest quality aphasia‐relevant stroke guideline [7] that includes seven recommendations and 10 practice points for aphasia services [31]. Recommendations were shortlisted based on the strength of research evidence, with Level I or II recommendations (per NHMRC evidence levels [32]) from the AARP and ‘strong’ recommendations (per GRADE [33]) from the Australian and New Zealand CGSM if considered relevant to speech pathologists' aphasia management practices. To ensure that recommendations important to people with lived experience of aphasia were represented in the shortlist, recommendations from the consumer‐validated Aphasia United Best Practice Recommendations [34] and Australian consumer‐important recommendations [35] were added if not already included. This resulted in a shortlist of aphasia guideline recommendations that were evidence‐based and/or ‘consumer‐important’.

### 2.3 Phase 1b: Two‐Round e‐Delphi

The e‐Delphi study was conducted in alignment with the Recommendations for the Conducting and Reporting of Delphi Studies (CREDES) [36]. People with lived experience (i.e., people with aphasia and their families living in Australia) and health professionals (such as clinicians and researchers working in aphasia within Australia) were recruited through aphasia organisations and professional networks (e.g., the Centre for Research Excellence in Aphasia Rehabilitation and Recovery community of practice, the Queensland Aphasia Research Centre and the Australian Aphasia Association). Snowball sampling was used, with potential participants encouraged to recruit other eligible participants from their networks. After completing Phase 1, participants were given the opportunity to continue in Phases 2 and/or 3.

#### 2.3.1 e‐Delphi Round 1: Perceived Importance of Recommendations

Participants with lived experience and healthcare professionals completed an online survey using Qualtrics [37]. The lived experience version was presented with accessible formatting and included video instructions, simplified language, graphics and voice recordings for each item. Participants individually rated their perceived importance of each shortlisted recommendation on a scale from 1 (*not important*) to 10 (*extremely important*) and then selected their top five priority recommendations.

*Analysis*. Survey data were exported to an Excel spreadsheet. Consistent with previous methods [27], the most important recommendations were identified by reviewing the median importance scores for each recommendation and the frequency with which each recommendation was ranked in the top five. Data were sorted to rank recommendations in order of importance (median, top five) for each participant group.

#### 2.3.2 e‐Delphi Round 2: Relative Importance and Implementation Feasibility

Participants ranked the relative importance of the recommendations identified in Round 1 in order from most important to least important. Health professional participants also ranked the recommendations in terms of relative feasibility for implementation in response to the question, ‘Which recommendations would be easiest to implement in clinical practice?’.

*Analysis*. Data were managed as per Round 1. The relative ranking of recommendations was compared by aggregating each participant's ranked responses into a ranked list for the whole group using a graph theory–based voting system implemented as a decision support tool in Microsoft Excel [38].

### 2.4 Phase 2: Quality Indicators for Routine Measurement (Consensus Meetings)

All participants from Phase 1 who had given permission to be contacted about subsequent phases were invited to participate in Phase 2. Purposive sampling was used to establish an expert panel representing key stakeholder groups (including people with lived experience, health professionals and researchers) and required expertise (in stroke/aphasia rehabilitation, epidemiology, health service evaluation and clinical and lived experience of aphasia). Shortlisted recommendations from the Phase 1 e‐Delphi were converted into quality indicators by the research team. Each indicator was defined and expressed with a numerator and denominator, a proposed information source and a timeframe for collection. Additional information including the strength of evidence, rationale for inclusion and information regarding acceptability and feasibility of routine collection was presented. Participants were provided with this information before the consensus meetings and people with lived experience received communication‐accessible versions.

A single 2‐h meeting was planned with each stakeholder group. Stakeholder groups met separately to ensure that sufficient time and resources could be allocated to communication support for lived experience experts. This approach also provided a means of mitigating potential power imbalances, which may have influenced participation. Two 2‐h meetings were ultimately required with the health professional and researcher group due to the large number of participants and lengthy discussions.

The health professional and researcher group met first. During these meetings, each indicator was presented alongside the supporting information outlined earlier and discussed.

Health professionals and researchers were asked to reflect on the following questions:

1. Is the indicator important and relevant?
2. Does the indicator measure the issue of interest?
3. Is there evidence for the use of the indicator in clinical practice or audit?
4. Is there scientific evidence that the indicator is associated with important health outcomes?
5. Is the indicator acceptable?
  a. Is there a sound clinical or empirical rationale for measuring it?
  b. Does it have meaning to consumers/clinicians/managers?
6. Is the indicator feasible to collect?
  a. Are the target population/exclusions well defined?
  b. Are data available from existing sources?

Each meeting was video‐recorded. After each meeting, key discussion points were summarised, and corresponding amendments were made to the indicators.

The lived experience group then met. The revised indicators and a summary of key discussion points were presented to the group in an accessible video presentation. People with lived experience were then asked to discuss each indicator and vote ‘yes’ or ‘no’ to the following questions:

1. Is the indicator important and relevant?
2. Does the indicator align with the recommendation?

Again, this meeting was video‐recorded. A summary of the lived experience groups' votes and discussions was then circulated back to the health professional and researcher group. With the knowledge of the lived experience groups' votes and discussions, the health professional and researcher group then voted on the indicators to determine if they should be piloted for routine measurement in aphasia services. A consensus was predefined as a vote of ‘yes’ by ≥ 70% of both stakeholder groups.

### 2.5 Phase 3: Priorities for Implementation (Priority‐Setting Workshop)

All participants from Phase 1 who had given permission to be contacted about subsequent phases were invited to participate in Phase 3. Participants (including people with lived experience and health professionals) were purposively sampled to ensure the representation of different clinical settings (acute, rehabilitation and community), funding models (public and private health), locations (metropolitan and regional), years of clinical experience for clinicians and different aphasia severity (mild, moderate and severe) for people with aphasia. Aphasia severity was a sampling consideration in this component as aphasia severity has been identified as a key implementation barrier to evidence‐based care [15]. Aphasia severity was independently rated using the Aphasia Severity Rating Scale [39] by two members of the research team (K.S. and S.J.W.). Participants from Phase 1 who were interested in Phase 3 were asked a series of sampling questions based on our sampling matrix, then further recruitment occurred as needed. Two additional speech pathologists were recruited who worked in rural areas in addition to one person with aphasia and their significant other.

A 2‐h online priority‐setting workshop was conducted to determine the top three implementation priorities for aphasia services. Before the workshop, participants were provided with a video summary of the project findings presented in lay terms. During the workshop, participants were presented with the recommendations identified as most important and most feasible. Each recommendation was discussed, and information was presented based on the following three criteria: (1) feasibility of change, (2) evidence‐practice gap and (3) meaningful impact. Participants were asked to judge the feasibility of services implementing the recommendations and changing practice within 6–12 months. Following this facilitated discussion, participants voted individually on their top three implementation priorities anonymously using a Google Forms survey, with points weighted for the priority selected (first priority = 3 points, second priority = 2 points, third priority = 1 point).

*Analysis*. Voting response data were extracted in Excel and summed, with priorities determined by the highest points.

## 3 Results

### 3.1 Phase 1a: Review and Prioritisation of Best Practice Recommendations

Following review of the AARP [8] and the CGSM [31] recommendations relevant to aphasia care, and consumer‐validated recommendations [34, 35], a shortlist of 23 best practice recommendations for aphasia were identified, as shown in Table 1.

**Table 1:** Responses to e‐Delphi Round 1 regarding the importance of best practice recommendations.

Recommendations				People with lived experience (*n* = 26)			Health professionals and researchers (*n* = 81)		
*Evidence‐based recommendations from Australian Aphasia Rehabilitation Pathway* [8] *or Australian and New Zealand Clinical Guidelines for Stroke Management* [31] 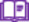			*Consumer‐important recommendations from Aphasia United Best Practice Statements* [34] *or Australian consumer‐important recommendations* [35] 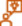	Frequency of being top five most important, *n* (%)	Median rating, *n*	Mean rating, *n*	Frequency of being top five most important, *n* (%)	Median rating, *n*	Mean rating, *n*
1		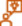	All patients with stroke should be **screened** for aphasia using a valid and reliable tool [34].	6 (23)	9	8.3	10 (12)	9	8.7
2		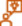	The person with suspected aphasia should be **assessed** by a speech pathologist to determine the presence and severity of aphasia [34].	13 (50)	10	9.3	33 (41)	10	9.7
3	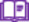	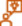	All people with aphasia should be **offered information** tailored to meet their changing individual needs using relevant language and communication formats. This should include information about the impact of aphasia and treatment options and use the word ‘aphasia’ [8, 31, 34, 35].	8 (31)	10	9.0	37 (46)	10	9.4
4		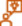	Speech pathologists should integrate information about **expected recovery and prognosis** into education and goal setting [35].	4 (15)	9	8.0	3 (4)	8	8.2
5	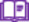	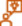	**Carers** should be given tailored **information and support** at all stages of recovery, including connection with appropriate social supports and support organisations [8, 31, 34].	8 (31)	9.5	8.2	7 (9)	10	9.2
6	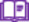	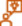	Aphasia rehabilitation should include **communication partner training to family, carers** and frequent communication partners of people with aphasia to improve communication and the communicative environment [8, 31, 34].	8 (31)	9	8.3	53 (65)	10	9.5
7	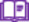	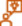	All **health and social care providers** working with people with aphasia across the continuum of care (i.e., acute care to end of life) should be **educated** about aphasia **and trained** to support communication in aphasia [8, 31, 34, 35].	7 (27)	10	9.2	21 (26)	10	9.4
8	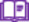		**Recovery goals should be set together** with the person with aphasia, their family or carer and speech pathologist. The goals should be well‐defined, specific and challenging, clearly documented and reviewed and updated regularly [31].	7 (27)	9	8.7	27 (33)	10	9.3
9		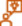	Aphasia services should be **person and family centred** [35]**.**	5 (19)	10	8.8	23 (28)	10	9.6
10	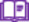	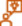	People with aphasia should be **offered therapy** to gain benefits in receptive and expressive language, and communication in everyday environments. This should include people with chronic aphasia who have ongoing goals [8, 31, 34].	5 (19)	10	9.3	28 (35)	10	9.4
11		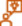	People with aphasia should receive **training, support and access to technology** for communication [35].	7 (27)	10	9.1	3 (4)	8	8.3
12	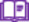	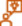	People with aphasia **earlier than 1 month** post‐onset could have **access to intensive aphasia rehabilitation** if they can tolerate it [8, 31, 34].	3 (12)	10	8.9	3 (4)	9	8.8
13	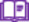	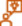	People with aphasia **after 1 month** should have **access to intensive aphasia rehabilitation** if they can tolerate it (at least 45 min of direct language therapy for 5 days a week) [8, 31, 34].	5 (19)	10	8.4	12 (15)	9	8.8
14	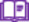	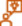	Aphasia rehabilitation should be **comprehensive and individualised** to address the impact of aphasia on functional everyday activities, participation and quality of life. This can include the impact upon relationships, vocation and leisure [8, 31, 34, 35].	8 (31)	10	9.6	51 (63)	10	9.5
15	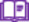	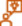	Speech pathologists should provide **evidence‐based treatment** for aphasia. Treatments with strong evidence include cognitive neuropsychology–based approaches and constraint‐induced language therapy [8, 34].	6 (23)	10	8.7	21 (26)	9	8.8
16	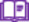	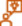	Speech pathologists should use effective aphasia service delivery approaches including **group therapy** and conversation groups [8, 34].	5 (19)	8	8.1	1 (1)	9	8.5
17	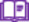	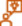	Speech pathologists should use effective aphasia service delivery approaches including **computer‐based treatments** [8, 34].	2 (8)	6	6.4	3 (4)	8	8.1
18	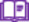		Speech pathologists should use effective aphasia service delivery approaches including **therapy provided by trained volunteers** [8].	0 (0)	6.5	6.4	1 (1)	8	7.9
19	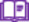	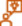	Where the speech pathologist is not proficient in a language of the person with aphasia, **a trained and qualified interpreter**, knowledgeable with the specific requirements for speech pathology, **should be used** [8, 34].	3 (12)	10	8.9	8 (10)	9	9.1
20		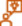	People with aphasia and their families should be **offered psychological support**. This includes support with depression, grief, loss and coping [35].	5 (19)	9.5	8.3	20 (25)	10	9.2
21	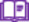	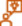	The speech pathologist should facilitate appropriate **social supports** for people with aphasia and their families, such as peer support, aphasia groups or support organisations [8, 35].	3 (12)	9	8.7	10 (12)	9	8.5
22	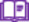		A **comprehensive discharge care plan** that addresses the patient's specific needs should be initiated early, developed together with the person with aphasia and their carer before hospital discharge, and shared with the receiving healthcare providers [8].	3 (12)	10	8.7	12 (15)	10	8.9
23		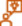	People with aphasia **should be supported to** build the skills needed to **self‐manage** their recovery and re‐integrate in their community [35].	4 (15)	10	9.3	18 (22)	9	9.1

*Note:* Dark grey indicates the most important recommendations identified by both groups, whereas light grey indicates highly‐rated recommendations (with the top 10) identified by one group and rated as extremely important (median of 10) by the other group. The bold text represents key words in the recommendations.

### 3.2 Phase 1b: e‐Delphi Round 1: Importance of Recommendations

Twenty‐six people with lived experience and 81 health professionals completed the Round 1 survey. The lived experience group included people with aphasia (*n* = 22) and significant others (*n* = 4); the majority were female (82%) with a median age of 57 years. The health professional group (*n* = 81) included 47 clinicians, 16 researchers and 18 clinician‐researchers. Ninety‐four percent had a speech pathology background, whereas 6% were from other healthcare disciplines (e.g., occupational therapy, neuropsychology, psychiatry and social work). Ninety‐eight percent of health professionals identified as female, with 38% in the 25–34 years age range.

From the survey of 23 items, the 10 ‘most important’ guideline recommendations were identified (Table 1). Overall, respondents indicated that all recommendations were important as the median ratings for health professionals and the lived experience group ranged from 8 to 10 and 6 to 10, respectively. Seven recommendations were rated by both participant groups as being ‘most important’ (see Table 1 for details—‘frequency of being selected as top five most important’). As no other recommendation was identified within the 10 ‘most important’ recommendations for *both* groups, the authorship team made the decision to include other highly‐rated recommendations if one group rated it as extremely important (i.e., median of 10). This allowed for the inclusion of three additional recommendations: ‘offering aphasia treatment’ (fifth most important for healthcare professionals, median score of 10 for lived experience group), ‘person and family centred services’ (seventh most important for healthcare professionals, median score of 10 for lived experience group) and ‘carer support’ (second most important for lived experience group, median score of 10 for healthcare professionals).

### 3.3 Phase 1b: e‐Delphi Round 2: Relative Importance and Feasibility

**Figure 2:**
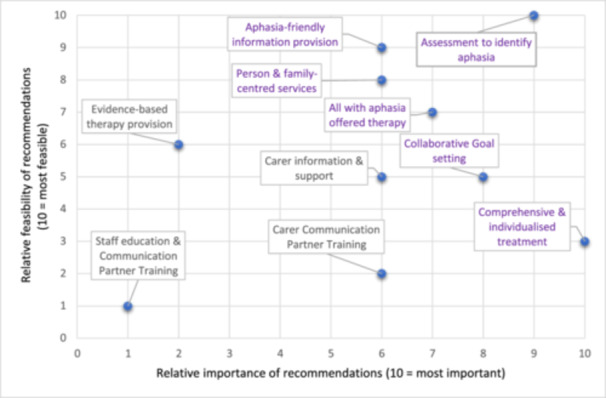
Responses to e‐Delphi Round 2 indicating relative importance and feasibility (10 = *most important/feasible*). Purple text indicates most important and feasible items. Items closer to the top right show higher aggregate rankings; closer to the bottom left are lower aggregate rankings; and top left and bottom right show items with higher scores in one dimension but not the other.

Seventeen people with lived experience and 49 health professionals responded to survey 2 (response rate = 81.5% of Round 1 participants) and ranked the top 10 recommendations. The recommendations, listed according to aggregate rankings of importance and feasibility per participant group, are presented in Table 2. There were considerable differences between the importance rankings per group, with half of the recommendations differing by three or more places (carer information and support, carer communication partner training, collaborative goal setting, person and family‐centred care and evidence‐based treatment). Given these different rankings and the larger size of the health professionals group, there was a risk that a ‘combined’ importance ranking would be dominated by health professional perspectives, diminishing the preferences of the lived experience group. Therefore, our research team made the pragmatic decision to use the importance rankings from the lived experience group and the feasibility rankings from the health professionals' group so both groups' preferences were represented; these are plotted in Figure 2 (scatter plot with importance on the horizontal axis and feasibility on the vertical axis). From these results, six recommendations were identified as being most important and most feasible for implementation.

**Table 2:** Responses to e‐Delphi Round 2 regarding relative importance and feasibility.

Ten most important best practice recommendations	Relative importance—lived experience ranking (*n* = 17) (10 = *highest*)	Relative importance—health professional ranking (*n* = 49) (10 = *highest*)	Relative feasibility—health professional ranking (*n* = 49) (10 = *highest*)
The person with suspected aphasia should be assessed by a speech pathologist to determine the presence and severity of aphasia.	9	10	10
All people with aphasia should be offered information tailored to meet their changing individual needs using relevant language and communication formats. This should include information about the impact of aphasia and treatment options and use the word ‘aphasia’.	6	7	9
Carers should be given tailored information and support at all stages of recovery, including connection with appropriate social supports and support organisations.	6	1	5
Aphasia rehabilitation should include communication partner training to family, carers and frequent communication partners of people with aphasia to improve communication and the communicative environment.	6	3	2
All health and social care providers working with people with aphasia across the continuum of care (i.e., acute care to end of life) should be educated about aphasia and trained to support communication in aphasia.	1	2	1
Recovery goals should be set together with the person with aphasia, their family or carer and speech pathologist. The goals should be well‐defined, specific and challenging, clearly documented and reviewed and updated regularly.	8	5	5
Aphasia services should be person and family centred.	6	9	8
People with aphasia should be offered therapy to gain benefits in receptive and expressive language, and communication in everyday environments. This should include people with chronic aphasia who have ongoing goals.	7	6	7
Aphasia rehabilitation should be comprehensive and individualised to address the impact of aphasia on functional everyday activities, participation and quality of life. This can include the impact upon relationships, vocation and leisure.	10	8	3
Speech pathologists should provide evidence‐based treatment for aphasia. Treatments with strong evidence include cognitive neuropsychology–based approaches and constraint‐induced language therapy.	2	5	6

### 3.4 Phase 2: Quality Indicators for Routine Measurement

The expert panel included 4 people with lived experience (3 people with aphasia, 2 significant other; 75% female) and 18 health professionals (7 clinicians, 11 researchers; 89% female) (see Supporting Information S1: File 1 for further detail). Some panel members were Phase 1 participants who expressed interest in continued participation in the project; however, additional recruitment also occurred to ensure the representation of stakeholder groups and increased breadth of expertise. The 10 shortlisted recommendations from Phase 1 were converted into 13 quality indicators for discussion and voting (Table 3). Across the two stakeholder groups, 11 quality indicators reached consensus to proceed to pilot testing for routine measurement in aphasia services (Table 3). Consensus was not reached for quality indicators relating to two recommendations: ‘comprehensive and individualised therapy’ and the ‘provision of evidence‐based treatment’, on the basis that they could not be practically operationalised for measurement in clinical practice. However, both indicators were considered important, and panel members felt they should be included if agreement on an appropriate definition could be reached.

**Table 3:** Results of quality indicator voting.

			Health professionals (*n* = 18)	People with lived experience (*n* = 4)		Both groups
Ten most important best practice recommendations		Quality indicator	*Indicator should be piloted for routine measurement* (%)	*Indicator is important and relevant* (%)	*Indicator aligns with recommendation* (%)	*Consensus reached*
1	The person with suspected aphasia should be assessed by a speech pathologist to determine the presence and severity of aphasia.	1a. A screener and/or assessment is completed to determine if communication impairment (including aphasia) is present.	100	100	100	Yes
		1b. A valid and reliable standardised assessment is conducted to determine the severity of aphasia.	83	100	100	Yes
2	All people with aphasia should be offered information tailored to meet their changing individual needs using relevant language and communication formats. This should include information about the impact of aphasia and treatment options and use the word ‘aphasia’.	2. Information about aphasia is provided to the person with aphasia.	71	100	100	Yes
3	Carers should be given tailored information and support at all stages of recovery, including connection with appropriate social supports and support organisations.	3a. Information about aphasia is provided to the person with aphasia's significant other(s).	89	100	100	Yes
		3b. Information about support is provided to the person with aphasia's significant other(s).	78	100	100	Yes
4	Aphasia rehabilitation should include communication partner training to family, carers and frequent communication partners of people with aphasia to improve communication and the communicative environment.	4. The primary communication partner of the person with aphasia is provided with communication partner training.	78	100	100	Yes
5	All health and social care providers working with people with aphasia across the continuum of care (i.e., acute care to end of life) should be educated about aphasia and trained to support communication in aphasia.	5a. Individualised recommendations for communicating with the person with aphasia are provided to the treating team.	83	100	100	Yes
		5b. There is training for staff in supported communication for aphasia.	83	100	100	Yes
6	Recovery goals should be set together with the person with aphasia, their family or carer and speech pathologist. The goals should be well‐defined, specific and challenging, clearly documented and reviewed and updated regularly.	6. Goal setting is undertaken in partnership with the person with aphasia and their significant others.	78	100	100	Yes
7	Aphasia services should be person and family centred.	7. The person with aphasia receives person/family centred care.	78	100	100	Yes
8	People with aphasia should be offered therapy to gain benefits in receptive and expressive language, and communication in everyday environments. This should include people with chronic aphasia who have ongoing goals.	8. The person with aphasia receives speech and language therapy.	83	100	100	Yes
9	Aphasia rehabilitation should be comprehensive and individualised to address the impact of aphasia on functional everyday activities, participation and quality of life. This can include the impact upon relationships, vocation and leisure.	9. Aphasia therapy is linked to goals.	67	100	100	No
10	Speech pathologists should provide evidence‐based treatment for aphasia. Treatments with strong evidence include cognitive neuropsychology–based approaches and constraint‐induced language therapy.	10. Speech pathologists who work with people with aphasia complete professional development in aphasia.	24	0	0	No

*Note:* Grey items did not reach consensus.

### 3.5 Phase 3: Priorities for Implementation

Five people with lived experience participated in the priority‐setting workshop, including four people with aphasia (50% with mild aphasia and 50% with moderate aphasia) and one significant other. Seven clinical speech pathologists participated (100% female; 71% with more than 10 years of clinical experience), representing a variety of clinical settings (71% rehabilitation), locations (57% metropolitan) and funding structures (86% public health); additional details are shown in Supporting Information S1: File 2. Participants discussed the six most important and feasible recommendations against implementation criteria and individually voted on their top three priorities for implementation. Non‐voting research members of the group facilitated the discussion. Table 4 shows results from the anonymous voting and the top three prioritised recommendations for implementation in aphasia services:

1. Assessment of aphasia.
2. Provision of aphasia‐friendly information.
3. Provision of therapy for people with ongoing goals.

**Table 4:** Results from anonymous voting on implementation priorities.

Shortlisted important and feasible recommendations	**First priority voting = 3 points,** *n* (%); **score**	**Second priority voting = 2 points,** *n* (%); **score**	**Third priority voting = 1 point,** *n* (%); **score**	Total score
The person with suspected aphasia should be assessed by a speech pathologist to determine the presence and severity of aphasia.	7 (58); 21	2 (17); 4	0	25
All people with aphasia should be offered information tailored to meet their changing individual needs using relevant language and communication formats. This should include information about the impact of aphasia and treatment options and use the word ‘aphasia’.	2 (17); 6	7 (58); 14	1 (8); 1	21
Recovery goals should be set together with the person with aphasia, their family or carer and speech pathologist. The goals should be well‐defined, specific and challenging, clearly documented and reviewed and updated regularly.	1 (8); 3	1 (8); 2	0	5
Aphasia services should be person and family centred.	1 (8); 3	0	4 (33); 4	7
People with aphasia should be offered therapy to gain benefits in receptive and expressive language, and communication in everyday environments. This should include people with chronic aphasia who have ongoing goals.	1 (8); 3	2 (17); 4	3 (25); 3	10
Aphasia rehabilitation should be comprehensive and individualised to address the impact of aphasia on functional everyday activities, participation and quality of life. This can include the impact upon relationships, vocation and leisure.	0	0	4 (33); 4	4

*Note:* Grey items were not voted as priorities.

## 4 Discussion

Through a rigorous and collaborative process, we have identified a minimum data set of 11 quality indicators for routine measurement and 3 implementation priorities in post‐stroke aphasia care. To our knowledge, this is the first research to include the preferences of people with lived experience of aphasia in the development of quality indicators and implementation priorities. The inclusion of multiple stakeholder groups and the use of an adapted criteria‐based prioritisation approach [9, 27] enabled indicators and priorities to be established concurrently. Our process was feasible and efficient, emphasising the value of obtaining multiple perspectives across multiple aspects of care.

Completion of a screener and/or assessment to determine the presence of aphasia achieved the highest level of agreement as an indicator of post‐stroke aphasia service quality. Unanimous agreement on this item likely reflects the recognition that high‐quality aphasia care is contingent on identification and referral. Although there are many aphasia screening tools [40, 41], most are designed for administration by a speech pathologist. A brief screening tool that can be administered by the multidisciplinary team is needed to support the early identification of communication impairment and appropriate and timely referral to speech pathology services. Documentation of screening in medical records is feasible and can be easily audited.

A novel and important component of this work was the inclusion of lived experience preferences from the first phase of identifying ‘evidence‐based’ recommendations. In the past, the starting point for prioritising recommendations for implementation was selecting those with high levels of research evidence [9, 27] before seeking end‐user input. By default, this has meant that recommendations with lower levels of research evidence such as qualitative studies were omitted and, therefore, may not have reflected consumer priorities. This was recently demonstrated where people with lived experience of aphasia proposed additional ‘consumer‐important’ recommendations [35] not present within the Aphasia United Best Practice Statements [34]. By incorporating these additional recommendations in our initial shortlist, five recommendations were included that had not been identified through filtering for high‐level ‘evidence‐based’ recommendations. The fact that at least one ‘consumer‐important’ recommendation (‘Aphasia services should be person and family centred’) was retained throughout the e‐Delphi surveys and Phase 2 consensus process reinforces the value of prospectively including perspectives of people with lived experience. This has implications for others seeking to identify quality indicators and implementation priorities in healthcare, suggesting the need to validate best practice recommendations through direct stakeholder engagement before prioritising recommendations where possible.

Although 7 recommendations were within the top 10 most important of both stakeholder groups, there were nonetheless clear differences in rankings. In survey 1b, recommendations relating to carer support, Communication Partner Training (CPT) for family members and collaborative goal setting, were ranked three of more places higher (i.e., as more important) by the lived experience group than the health professional group. It should be noted that these three recommendations relate to the aspects of carer involvement in aphasia services, likely reflecting that there are ongoing unmet needs in this area [42]. There is a history of divergent opinions between clinical speech pathologists and people with lived experience of aphasia in a number of areas including rehabilitation goals [43], outcomes [44] and recovery [45], so it is not surprising that stakeholder groups had different perspectives in our study. This further reinforces the need to respond to differences throughout prioritisation studies to ensure that the representation of these views is maintained.

Three priorities were identified as implementation targets for aphasia services within the Australian context: assessment of aphasia, provision of aphasia‐friendly information and provision of therapy for people with ongoing goals. The ‘aphasia‐friendly information provision’ topic is consistent with implementation priorities identified previously in aphasia [9] and the broader stroke population [27]. The continued presence of information provision as a priority reflects ongoing implementation challenges in this area [12, 15]. Similarly, concepts related to the umbrella topic of ‘provision of therapy’ such as therapy dose and specific treatment approaches were previously identified as aphasia implementation priorities [9]. However, the ‘assessment’ of aphasia topic has not previously been prioritised, as it was not included in the initial shortlist of recommendations [9] due to relatively low levels of evidence. The identification of ‘assessment’ as the highest priority further supports the benefit of adapting established implementation criteria to incorporate consumer‐important recommendations.

All three implementation priorities were ranked as moderately or highly feasible. Although there is currently no systematic method to assess feasibility of implementing recommendations [46], the perception of ‘low’ feasibility in our study appeared to reflect recommendations requiring multidisciplinary input or delivery of complex interventions. For example, CPT, a complex intervention requiring involvement of healthcare staff and/or family members [47], was ranked the least feasible of the top 10 recommendations. This low feasibility ranking has implications for delivering communication partner training in clinical settings and aligns with previously identified implementation challenges such as perceived willingness of communication partners to be involved [14]. Further work informed by implementation science is needed to understand how to overcome existing barriers for more ‘complex’ recommendations such as CPT to enhance feasibility of implementation.

It is important to note that for half (3/6) of the recommendations considered within the prioritisation workshop, no data were available to quantify any ‘evidence‐practice’ gap, and none of these six recommendations were measured routinely. Seeing as there is limited routine measurement of aphasia practices [11], the decision to include recommendations regardless of whether they were currently being measured was considered a necessary adaption of previous prioritisation criteria [27]. If inclusion had been restricted to those recommendations being measured and not being delivered as intended, then very few recommendations would have been included in the initial shortlist. This may have implications for future implementation and quality improvement efforts, as implementation strategies can only be evaluated if the performance of the intended practice is measured [48].

## 5 Implications and Future Directions

Our adapted approach of combining research evidence, the perspectives of people with lived experience and pre‐determined implementation criteria is unique to the field of aphasia. Public and patient involvement in this process ensured an equal voice in voting and prioritisation. Therefore, our findings have a powerful authority that should instil services with confidence to adopt identified quality indicators and implementation priorities.

Interestingly, the three priorities reflect key aspects of aphasia management from the diagnosis of aphasia through assessment, provision of information to patients and families and provision of therapy to people with ongoing goals. The prioritisation of these areas may reflect a desire for more consistency in the ‘basics’ of aphasia care before addressing areas perceived as being more complex, multidisciplinary or more specific (e.g., minutes of therapy delivered as an indication of treatment dose). We encourage services to reflect on their practice in these areas. Importantly, our adapted prioritisation process can serve as a model for other areas of healthcare that, similar to aphasia, have limited routine data collection and where there may be a lack of strong evidence‐based guideline recommendations.

We encountered several challenges in operationalising and gaining consensus on the quality indicators. One challenge was the process of converting each recommendation into a measurable quality indicator. For example, it was difficult to operationalise the multifactorial concept of person‐centred care, as this is an ongoing philosophy of care that cannot easily be gauged at any one time point. Another challenge during this process was considering the availability of clinical data and the feasibility of collecting it in clinical practice. As a next step to address this challenge, our research team has obtained funding to pilot the agreed quality indicators. An embedded process evaluation component will explore the feasibility and acceptability of collecting the quality indicators in clinical practice from the perspectives of speech pathologists and people with aphasia.

## 6 Limitations

Despite best efforts, there were limitations to our inclusion of people with lived experience across the phases of research. For example, in the e‐Delphi, there were differences in stakeholder group size with smaller numbers of people with lived experience compared to healthcare professionals. We responded to this limitation by being cognisant of differences in representation and adapted our approach when needed to ensure that the voices of different stakeholder groups were balanced. Additionally, it would have been preferable to have people with lived experience of aphasia on our project team to guide recruitment and provide feedback on whether lived experience viewpoints were adequately represented.

Although international research informed the guidelines and best practice statements used in this study, the proposed indicators and priorities are applied to the Australian healthcare context and may lack generalisability elsewhere. Another potential limitation was that the ‘most important’ recommendations used to inform the quality indicators and implementation priorities are representative of one time period. We acknowledge that stakeholder perceptions of importance may change and will therefore need to be reviewed and updated in future. Despite these limitations, we now have a starting point to improve aphasia services within the Australian context.

## 7 Conclusion

There was a consensus on 11 quality indicators and 3 implementation priorities for aphasia services through a combination of lived, clinical and research expertise. The quality indicators are the necessary first step to enabling efficient and person‐centred service improvement efforts. These will be embedded in routine data collection systems. In the future, national implementation strategies will address the three priority areas to deliver the greatest gains for Australian stroke survivors with aphasia.

## Author Contributions

**Kirstine Shrubsole:** conceptualisation, funding acquisition, writing–original draft, methodology, formal analysis, investigation, project administration, supervision. **Marissa Stone:** writing–review and editing, writing–original draft. **Dominique A. Cadilhac:** writing–review and editing, methodology. **Monique F. Kilkenny:** methodology, writing–review and editing. **Emma Power:** conceptualisation, investigation, writing–review and editing, methodology, formal analysis. **Elizabeth Lynch:** conceptualisation, writing–review and editing, methodology, formal analysis. **John E. Pierce:** conceptualisation, writing–review and editing, methodology, formal analysis, visualisation. **David A. Copland:** writing–review and editing. **Erin Godecke:** writing–review and editing. **Bridget Burton:** writing–review and editing. **Emily Brogan:** writing–review and editing. **Sarah J. Wallace:** conceptualisation, investigation, writing–original draft, project administration, methodology, supervision.

## Ethics Statement

The study was approved by a Human Research Ethics Committee.

## Conflicts of Interest

Authors M.S., D.A.C., M.K., E.P., E.L., J.E.P., D.A.C., E.G., and E.G. were involved in the Phase 2 expert panel, listed in Supporting Information S1: File 1. The other authors declare no conflicts of interest.

## Supporting information

Supporting information.

## Data Availability

The data that support the findings of this study are available from the corresponding author upon reasonable request.
