# Supplementary material for: Establishing Quality Indicators and Implementation Priorities for Post‐Stroke Aphasia Services Through End‐User Involvement
Source: Health Expect. 2024 Sep 2;27(5):e14173. doi: 10.1111/hex.14173 (PMC11369030; doi:10.1111/hex.14173)
Supplement: Supplementary file 1 — Supporting information. [file HEX-27-e14173-s001.docx]

*Supplementary File 1. Phase 2 meeting facilitators and expert panel*

*Meeting facilitators*

| **Sarah J. Wallace, PhD**  *Background: Principal research fellow in aphasia rehabilitation and clinical speech pathologist* | **Kirstine Shrubsole, PhD**  *Background: Research fellow in aphasia rehabilitation and clinical speech pathologist* |
| --- | --- |

*Expert panel (Lived Experience)*

| **Julia Druery**  Family member of person with aphasia  *Background:* *Consumer Advisory Group member,* *Queensland Aphasia Research Centre* | **Kent Druery**  Person living with aphasia  *Background:* *Consumer Advisory Group member,* *Queensland Aphasia Research Centre* | **Kim Barron**  Person living with aphasia  *Background:* *Senior research assistant,* *Queensland Aphasia Research Centre* | **Parichat Srikaew**  Person living with aphasia |
| --- | --- | --- | --- |

*Expert panel (Health Professionals and Clinicians)*

| **Claire Bennington**  *Background: Clinical speech pathologist and doctoral research student in aphasia rehabilitation* | **Emily Brogan, PhD**  *Background: Clinical speech pathologist and conjoint research fellow in speech pathology* | **Dominique Cadilhac, PhD**  *Background: Professor and Australian Stroke Clinical Registry (AuSCR) Principal Investigator* | **David A. Copland, PhD**  *Background: Professor of speech pathology and Director of Queensland Aphasia Research Centre* |
| --- | --- | --- | --- |
| **Deborah Cross**  *Background: Clinical speech pathologist* | **Erin Godecke, PhD**  *Background: Associate Professor of speech pathology with research expertise in aphasia rehabilitation* | **Monique Kilkenny, PhD**  *Background: Associate Professor in epidemiology and Head of the National Stroke Data Linkage Program at Monash University* | **Elizabeth Lynch, PhD**  *Background: Senior research fellow with research expertise in stroke rehabilitation* |
| **Marie-Pier McSween, PhD**  *Background: clinical speech pathologist and postdoctoral research fellow in aphasia rehabilitation* | **Lindsey Nickels, PhD**  *Background: Professor of speech pathology with research expertise in aphasia rehabilitation* | **John E. Pierce, PhD**  *Background: clinical speech pathologist and research fellow in aphasia rehabilitation* | **Emma Power, PhD**  *Background: Associate Professor of speech pathology with research expertise in aphasia rehabilitation* |
| **Kylie Short**  *Background: clinical speech pathologist and Director of speech pathology and audiology* | **Chloe Smith**  *Background: clinical speech pathologist* | **Marissa Stone**  *Background: clinical speech pathologist and doctoral research student in aphasia rehabilitation* | **Helen Wallace**  *Background: clinical speech pathologist and doctoral research student in aphasia rehabilitation* |
| **Linda Worrall, PhD**  *Background: Professor of speech pathology and Director of Queensland Aphasia Research Centre* | **Sally Zingelman**  *Background: clinical speech pathologist and doctoral research student in aphasia rehabilitation* |  |  |

*Supplementary File 2. Phase 3 priority setting workshop facilitators and participant characteristics*

S2 Table 1. Workshop facilitators

| **Kirstine Shrubsole, PhD**  *Background: Research fellow in aphasia rehabilitation and clinical speech pathologist* | **Emma Power, PhD**  *Background: Associate Professor of speech pathology with research expertise in aphasia rehabilitation* | **Sarah J. Wallace, PhD**  *Background: Principal research fellow in aphasia rehabilitation and clinical speech pathologist* | **John E. Pierce, PhD**  *Background: Research fellow in aphasia rehabilitation and clinical speech pathologist* |
| --- | --- | --- | --- |
| **Elizabeth Lynch, PhD**  *Background: Senior research fellow with research expertise in stroke rehabilitation* |  |  |  |

S2 Table 2. Characteristics of clinical speech pathologist participants (n=7) according to sampling matrix

| **Years of clinical experience** | | **Setting/Role** **may have selected more than 1 setting* | | **Location** | | **Funding** | |
| --- | --- | --- | --- | --- | --- | --- | --- |
| < 10 | 2 (29%) | Acute | 4 (57%) | Metropolitan | 4 (57%) | Public | 6 (86%) |
| 10+ | 5 (71%) | Subacute/ Community Rehabilitation | 5 (71%) | Regional | 3 (43%) | Private | 1 (14%) |
|  |  | Community | 1 (14%) |  |  |  |  |
|  |  | Manager | 1 (14%) |  |  |  |  |

S2 Table 3. Aphasia Severity for Lived Experience Participants with Aphasia

| *Aphasia Severity per Aphasia Severity Rating Scale; Goodglass et al., 2001* | *People with aphasia (n=4)* |
| --- | --- |
| **Mild (rating of 3 or 4)** | **2** |
| **Moderate (rating of 2)** | **2** |
| **Severe (rating of 1)** | **0** |
